# Supplementary material for: A stationary phase-specific bacterial green light sensor for enhancing metabolite production
Source: Nat Commun. 2025 Dec 24;17:1071. doi: 10.1038/s41467-025-67829-8 (PMC12852798; doi:10.1038/s41467-025-67829-8)
Supplement: Supplementary file 1 — Supplementary Information [file 41467_2025_67829_MOESM1_ESM.pdf]

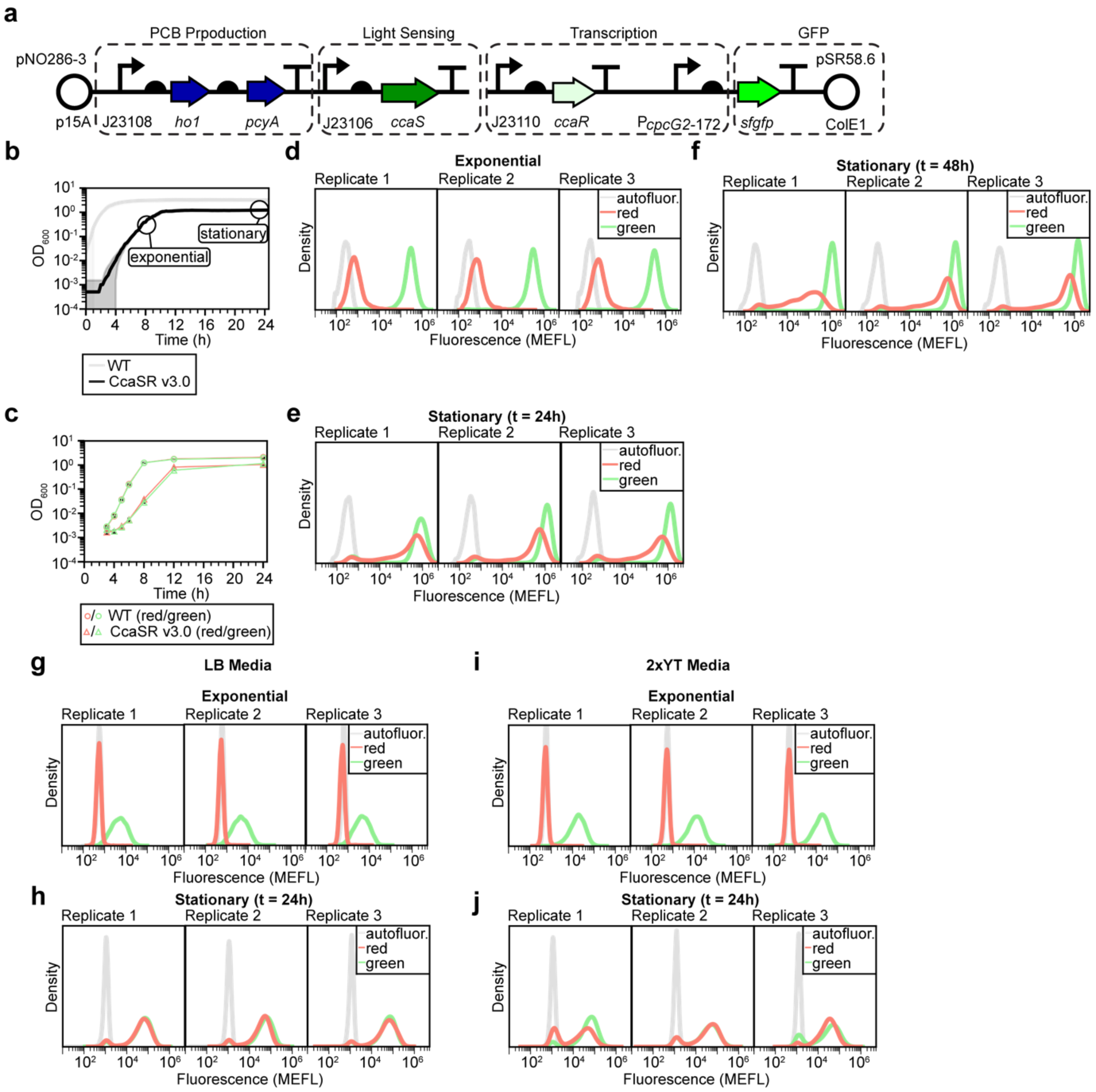

Supplementary Figure 1. Detailed characterization of CcaSR v3.0 in stationary phase. (a) Cartoon schematic of the 4 modules of CcaSR v3.0. (b) Growth curves of CcaSR v3.0 (black) and BW29655 (WT, gray). The gray shaded region represents the standard deviation of the mean of *n* = 3 independent biological replicates across three separate days. (c) Growth curves of CcaSR v3.0 and BW29655 in the presence of red/saturating green light. Error bars represent the standard deviation of the mean of *n* = 3 independent biological replicates collected on the same day. (d) Independent biological replicate histograms CcaSR v3.0:*sfgfp* strain fluorescence under

red and green light in exponential, (e) stationary phase (t = 24 h), and (f) stationary phase (t = 48 h). (g) Independent biological replicate histograms in LB media in both exponential and (h) stationary phase. (i) Independent biological replicate histograms in 2xYT media in both exponential and (j) stationary phase. All independent biological replicates were collected on the same day.

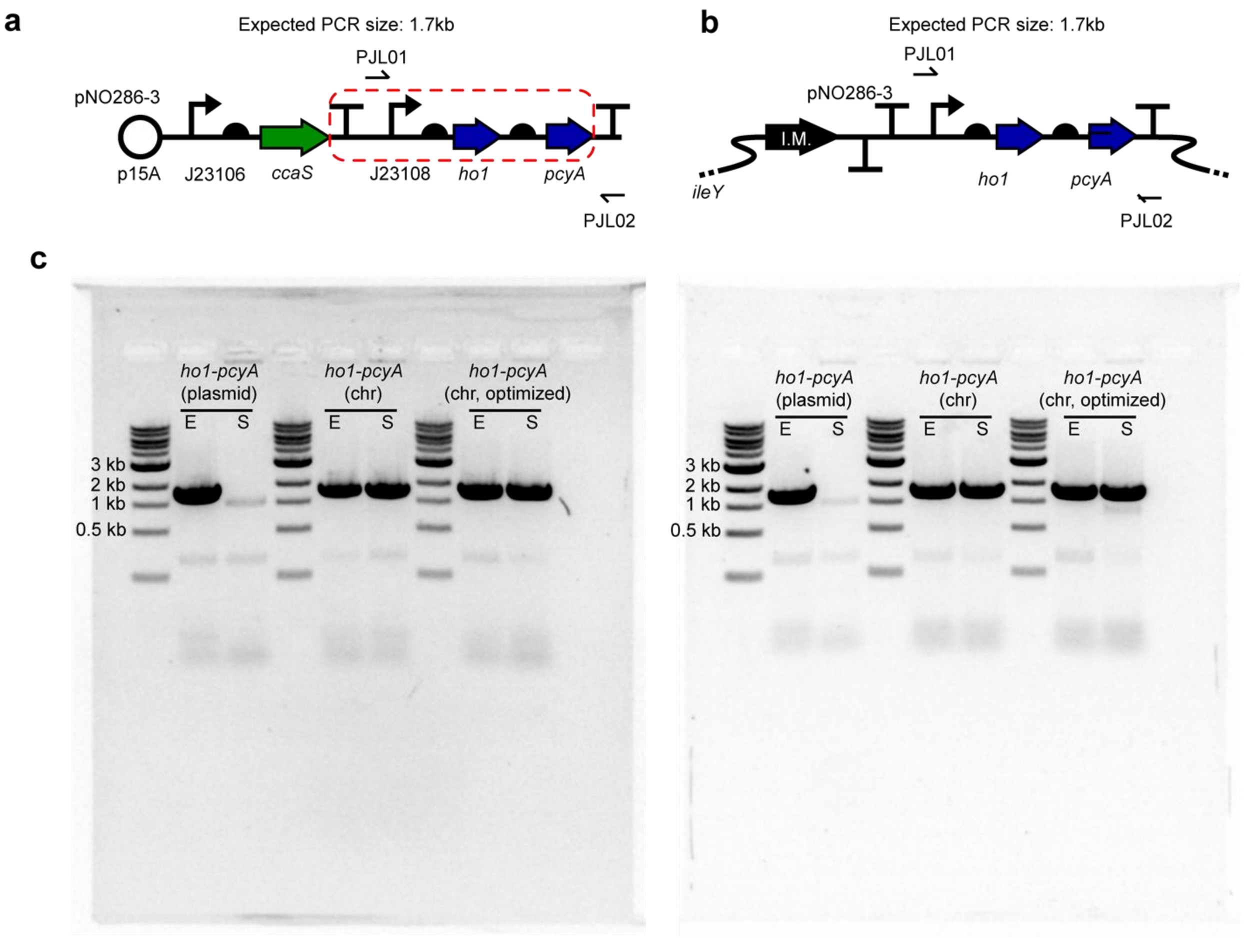

Supplementary Figure 2. Detailed diagnostic PCR of the *ho1-pcyA* cassettes. (a-b) Plasmid and chromosomal *ho1-pcyA* cassettes with diagnostic primer binding sites (horizontal lines with half arrowheads) in expected product size indicated. Red dotted box indicates the section of the plasmid that we found to be mutated in stationary phase. DNA sequencing results are provided in Supplemental Data 1. I.M: chromosomal integration module. (c) Full, unedited gel replicates of diagnostic PCRs from experiments on two separate days.

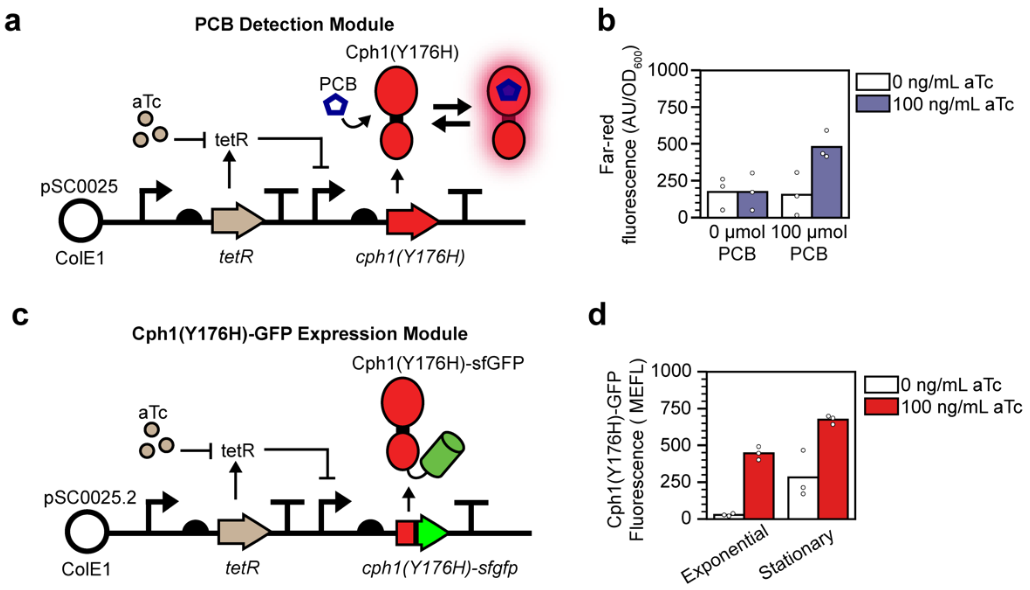

Supplementary Figure 3. Validation of Cph1(Y176H) PCB biosensor system in *E. coli.* (a) Diagram of PCB detection module. *cph1(Y176H)* expression is induced with anhydrotetracycline (aTc). (b) Detection of exogenously-supplied PCB (Fisher 50-145-5760) via induced Cph1(Y176H). Bacteria were diluted to $OD_{600} = 10^{-5}$ in the presence/absence of 100 µmol PCB and grown for 8 h in 24-well plates before being assayed via a fluorescence plate reader. Far red fluorescence was normalized to OD_{600} to correct for differences in culture density. Bars represent the arithmetic mean of $n = 3$ biologically independent replicates collected over 3 separate days. (c) Diagram of system used to confirm Cph1(Y176H) expression. (d) Cph1(Y176H)-sfGFP fluorescence in exponential and stationary phase with and without the addition of aTc. Bars represent the arithmetic mean Cph1(Y176H)-sfGFP fluorescence of $n = 3$ independent biological samples collected on the same day.

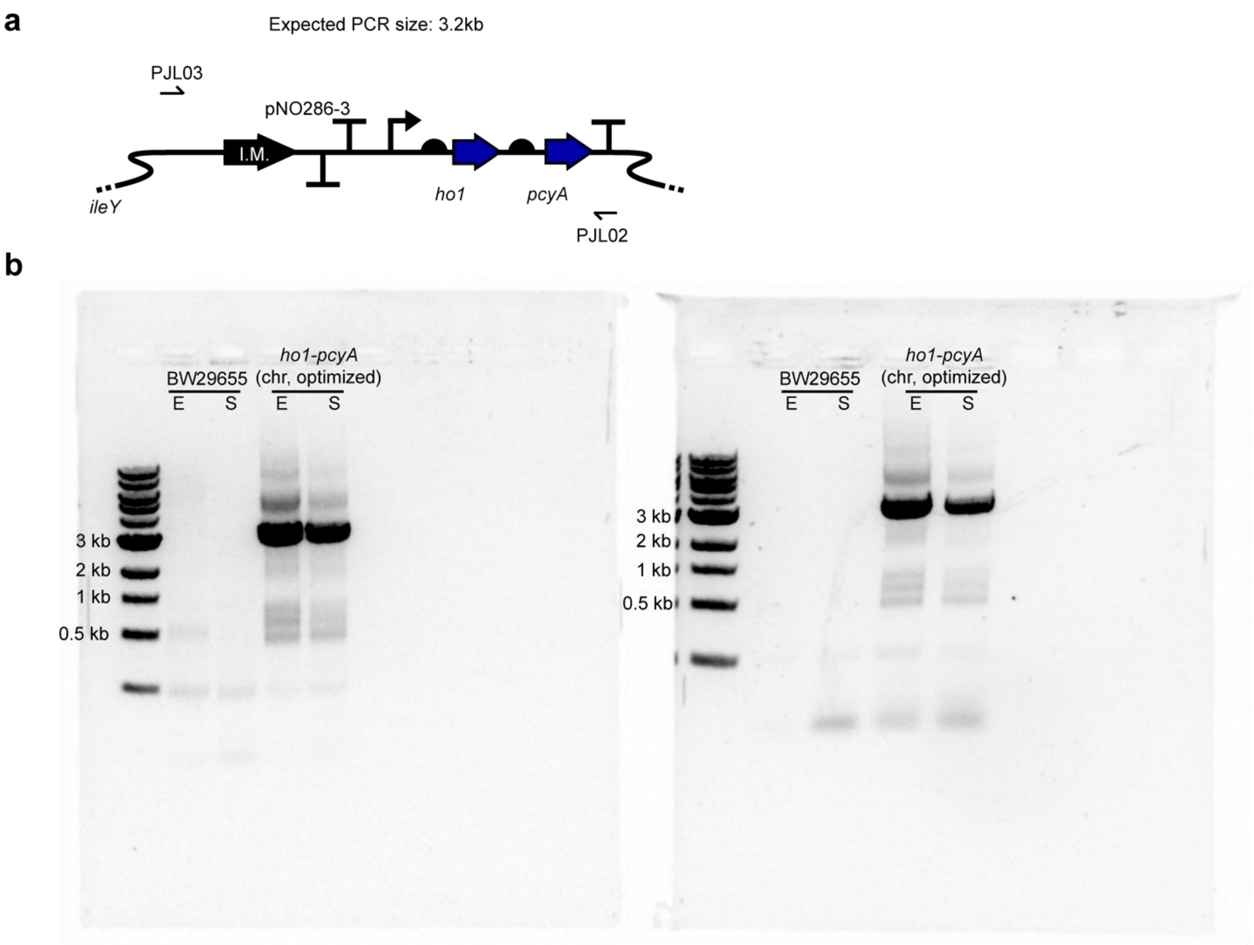

Supplementary Figure 4. Detailed diagnostic genomic PCR of the *ho1-pcyA* cassettes. (a) Chromosomal *ho1-pcyA* cassettes with a primer binding to the genome outside of the *ileY* locus (PJL03) and the integration cassette (PJL02). Expected product size indicated of successful amplification is indicated as 3.2kb. (b) Full, unedited gel replicates of diagnostic PCRs for both wild-type BW29655 and the optimized chromosomal integration cassette from experiments on two separate days.

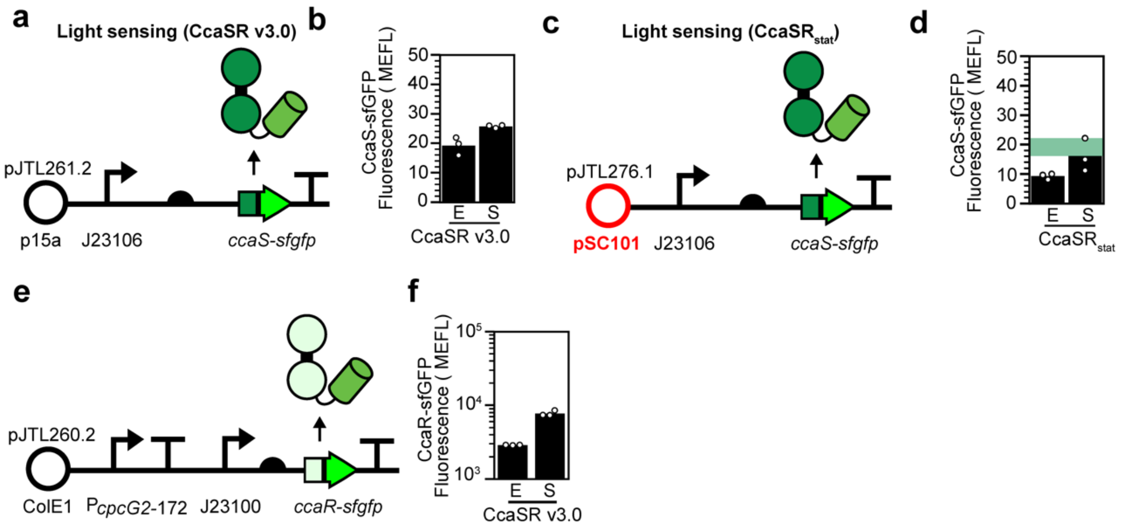

Supplementary Figure 5. CcaS and CcaR accumulate in stationary phase. GFP-tagging the sensor histidine kinase CcaS and the response regulator CcaR has been utilized in past literature as a method for roughly measuring expression of CcaS/CcaR[1]; thus we applied it for measuring CcaS/CcaR expression in exponential and stationary phase. (a) Diagram of *sfgfp*-tagged *ccaS* expression module corresponding to CcaSR v3.0. (b) Fluorescence of CcaS-sfGFP from cultures grown to exponential (E) and stationary (S) phase. Bars represent the mean CcaS-sfGFP fluorescence of n = 3 independent biological replicates collected on the same day. CcaS-sfGFP levels increase from 19.2 ± 3.0 MEFL in exponential phase to 25.7 ± 0.5 MEFL in stationary phase. Past studies with CcaSR have demonstrated that optimal expression ranges of *ccaS* are required for a high dynamic range[1,2]; thus, we aimed to reduce the stationary phase *ccaS* expression to be closer to that of its optimal exponential phase levels. (c) Diagram of *sfgfp*-tagged *ccaS* expression module corresponding to $CcaSR_{stat}$ where the ~9-copy p15a origin of replication[3] has been replaced with ~3-copy pSC101[3]. (d) Fluorescence of CcaS-GFP in both exponential and stationary phases of $CcaSR_{stat}$. Bars represent the mean CcaS-sfGFP fluorescence of *n* = 3 independent biological replicates collected on the same day. Solid

horizontal green shaded region represents the standard deviation of exponential phase fluorescence of CcaS-GFP in CcaSR v3.0. Stationary phase CcaS-GFP in $CcaSR_{stat}$ (16.1 ± 5.5 MEFL) is reduced to be near that of optimal exponential phase fluorescence of CcaS-GFP in CcaSR v3.0. (e) Diagram of a variant of pSR58.6 wherein *ccaR* is fused to *sfgfp*. (f) CcaR-sfGFP fluorescence in exponential and stationary phases. Bars represent the mean CcaR-sfGFP fluorescence of *n* = 3 independent biological replicates data points collected on the same day. CcaR-GFP fluorescence accumulates from 2,880 ± 20 MEFL in exponential phase to 7,750 ± 760 MEFL in stationary phase.

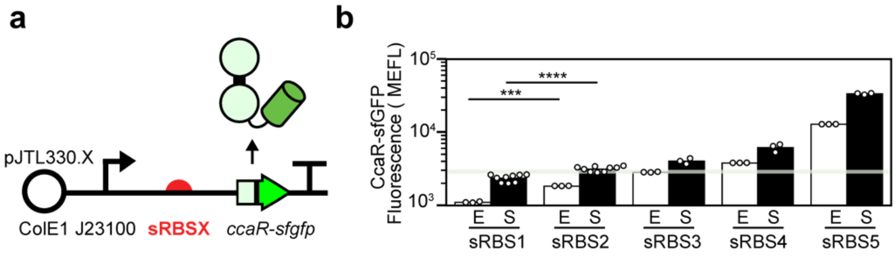

Supplementary Figure 6. Exponential and stationary phase expression of *ccaR* from different sRBSs. (a) *ccaR* tagged with *sfgfp* to evaluate strength of five sRBSs. (b) CcaR-sfGFP fluorescence of each strain in exponential and stationary phases. The horizontal line represents the mean of exponential phase CcaR-sfGFP expression in the context of the CcaSR v3.0 system (Supplementary Fig. S4D). Bars represent the mean CcaR-sfGFP fluorescence of at least $n = 3$ independent biological replicates data points collected on the same day. There exists a statistically significant change in *ccaR-sfGFP* expression between sRBS1 and sRBS2 in both exponential and stationary phase (two-tailed Student's t-test $p = 3 \times 10^{-4}$ and 3×10^{-5}, respectively).

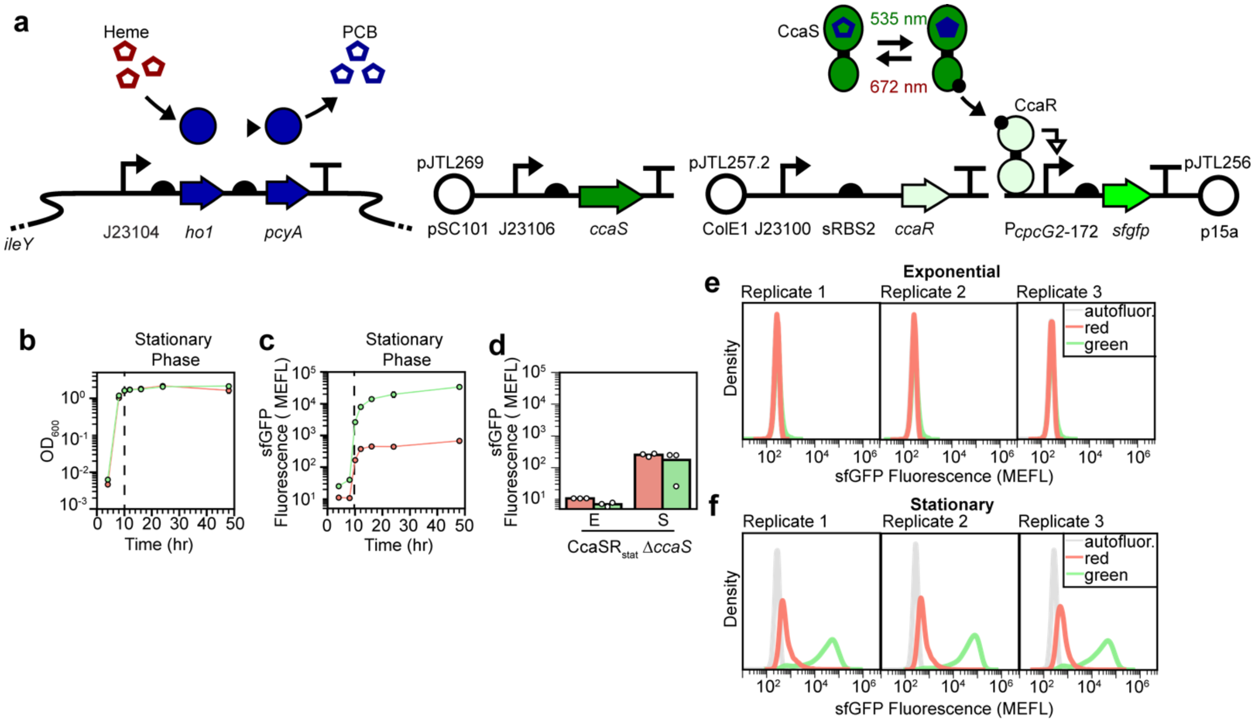

Supplementary Figure 7. CcaSR$_{stat}$ output in different growth phases. (a) CcaSR$_{stat}$:*sfgfp* system. (b) OD$_{600}$ and (c) sfGFP fluorescence of the CcaSR$_{stat}$:*sfgfp* strain in the presence of red and green light over 48 h of culture growth. The saturating intensity of 10 μmol m^{-2} s^{-1} was utilized for green light induction. Vertical dashed lines indicate the onset of stationary phase. Data points indicate the arithmetic mean GFP fluorescence. Error bars represent standard deviation of n = 3 independent biological replicates collected on the same day. (d) Light response of a CcaSR$_{stat}$ strain lacking *ccaS* (CcaSR$_{stat}$ Δ*ccaS*) in exponential and stationary phases. (e) Histograms of the CcaSR$_{stat}$:*sfgfp* strain fluorescence in red and green light in both exponential (t = 8 h) and (f) stationary (t = 24 h) phase of the independent biological samples collected on the same day.

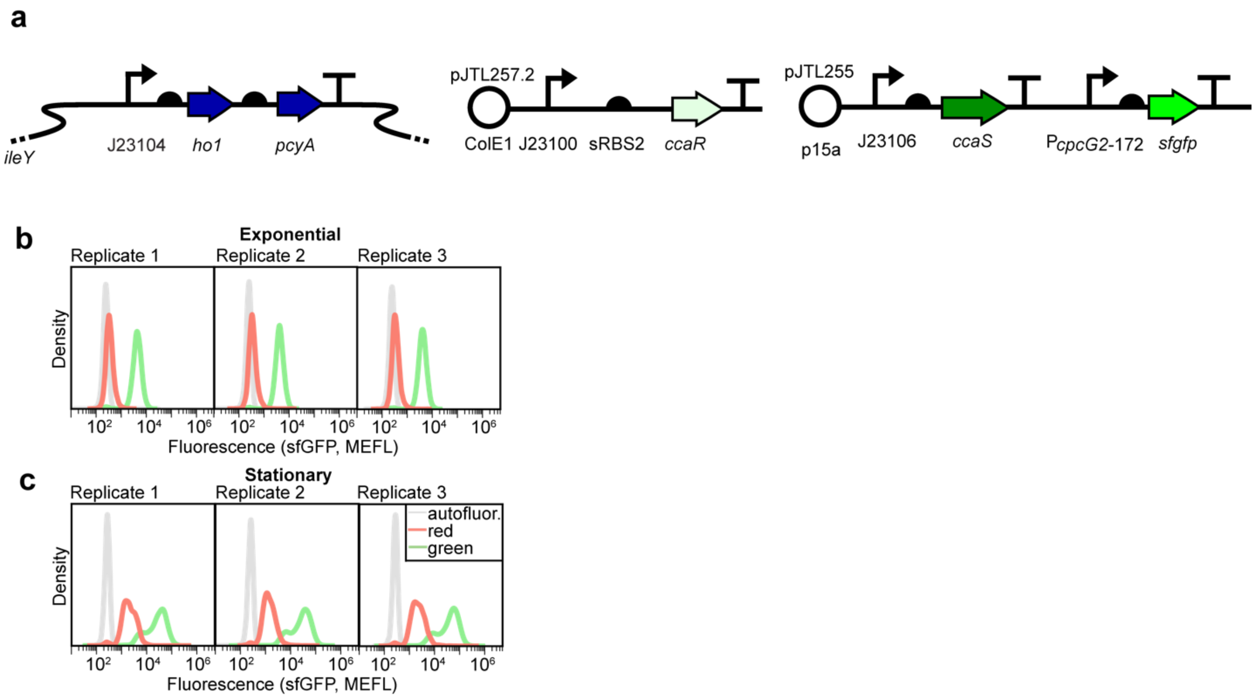

Supplementary Figure 8. A CcaSR$_{\mathrm{stat}}$ variant with increased *ccaS* expression functions in exponential and stationary phase. (a) CcaSR$_{\mathrm{stat}}$ modified to encode the *ccaS* cassette on a higher copy p15a plasmid, as in the original CcaSR v3.0 system. (b) Fluorescence histograms of the modified CcaSR$_{\mathrm{stat}}$ strain in red and green light in both exponential and (c) stationary phase. Replicates were n = 3 independent biological experiments performed on the same day.

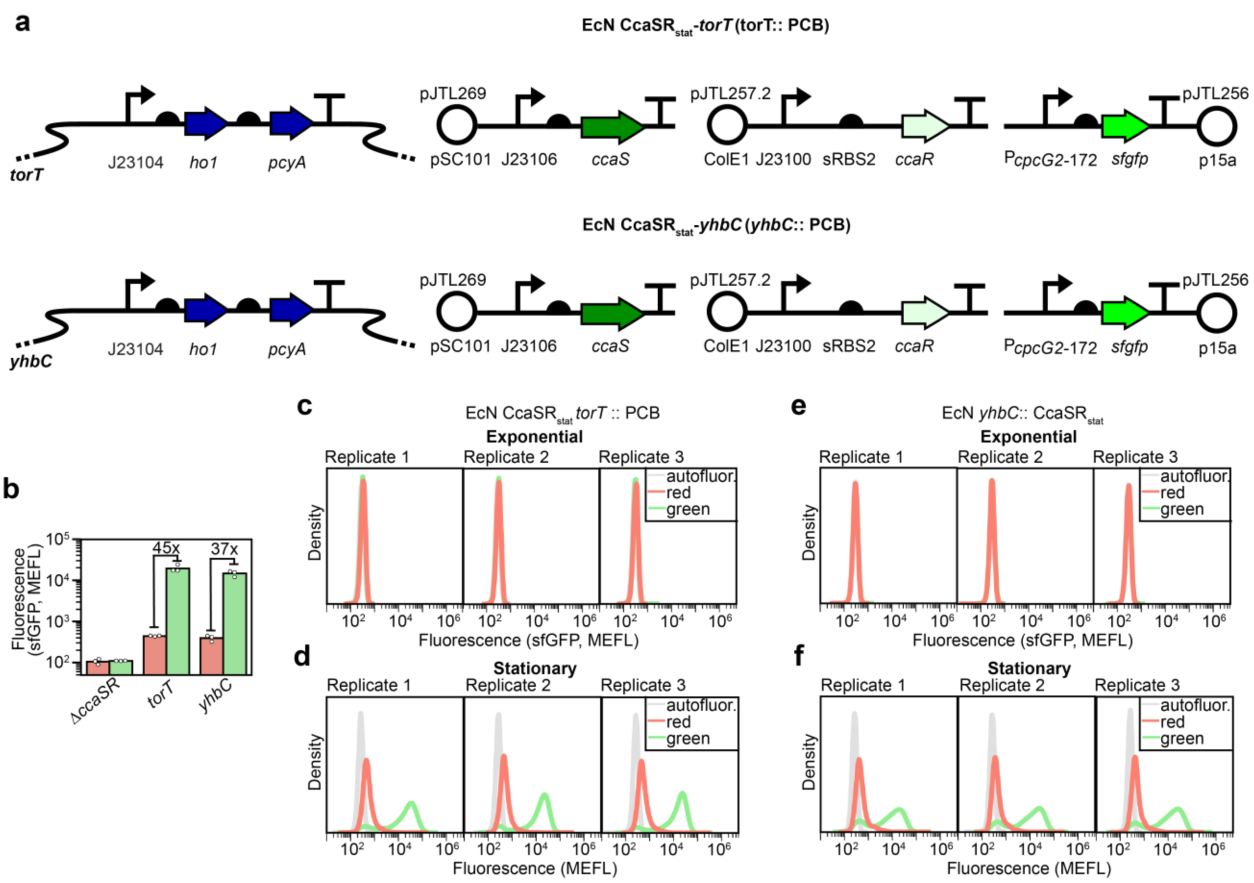

Supplementary Figure 9. Engineering CcaSR$_{stat}$ to function in *E. coli* Nissle 1917. (a) CcaSR$_{stat}$ schematics in *E. coli* Nissle 1917 (EcN). Strains EcN CcaSR$_{stat}$-*torT* and CcaSR$_{stat}$-*yhbC* have the *ho1-pcyA* expression cassette integrated into the *torT* and *yhbC* loci, respectively. (b) Light response of the EcN Δ*ccaSR* control (pJTL256 transformed into EcN) and the two EcN CcaSR$_{stat}$ strains in saturating red and green light. Bar plots represent the mean sfGFP fluorescence of 3 independent biological replicates collected on the same day. (c) Three independent biological replicate histograms of EcN *torT* :: CcaSR$_{stat}$ in exponential and (d) stationary phase collected on the same day. (e) Three independent biological replicate histograms of EcN *yhbC* :: CcaSR$_{stat}$ in exponential and (f) stationary phase collected on the same day.

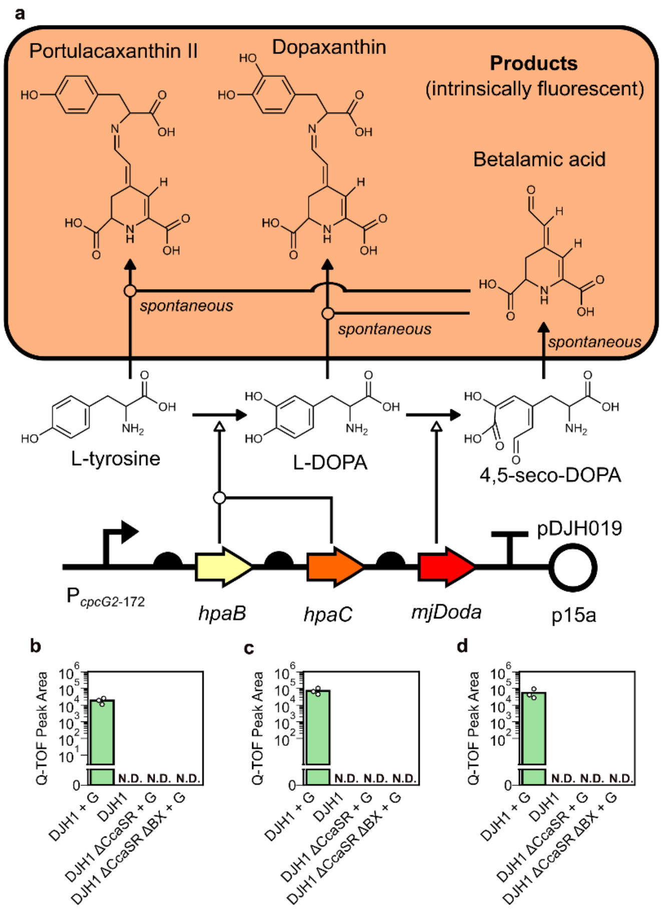

Supplementary Figure 10. CcaSR$_{stat}$-regulated betaxanthin pathway. (a) Engineered metabolic pathway for the production of betaxanthin pigments from L-tyrosine. (b) Q-TOF LC-MS peak areas of betalamic acid, (c) dopaxanthin, and (d) portulacaxanthin II. Conditions and strains tested are the full betaxanthin production strain DJH1 under 100 µmol m^{-2} s^{-1} green light (DJH1 + G), DJH1 with no green light (DJH1), DJH1 with the pDJH019 plasmid bearing the betaxanthin pathway (pDJH019) but lacking CcaSR (pJTL257.2, pJTL269) under green light (DJH1 ΔCcaSR + G), and DJH1 lacking pDJH019, pJTL257.2, and pJTL269 under green light (DJH1 ΔCcaSR ΔBX + G). N.D. indicates that a peak corresponding to the metabolite could not be detected with confidence. Bar graphs 3 biological replicates collected on the same day.

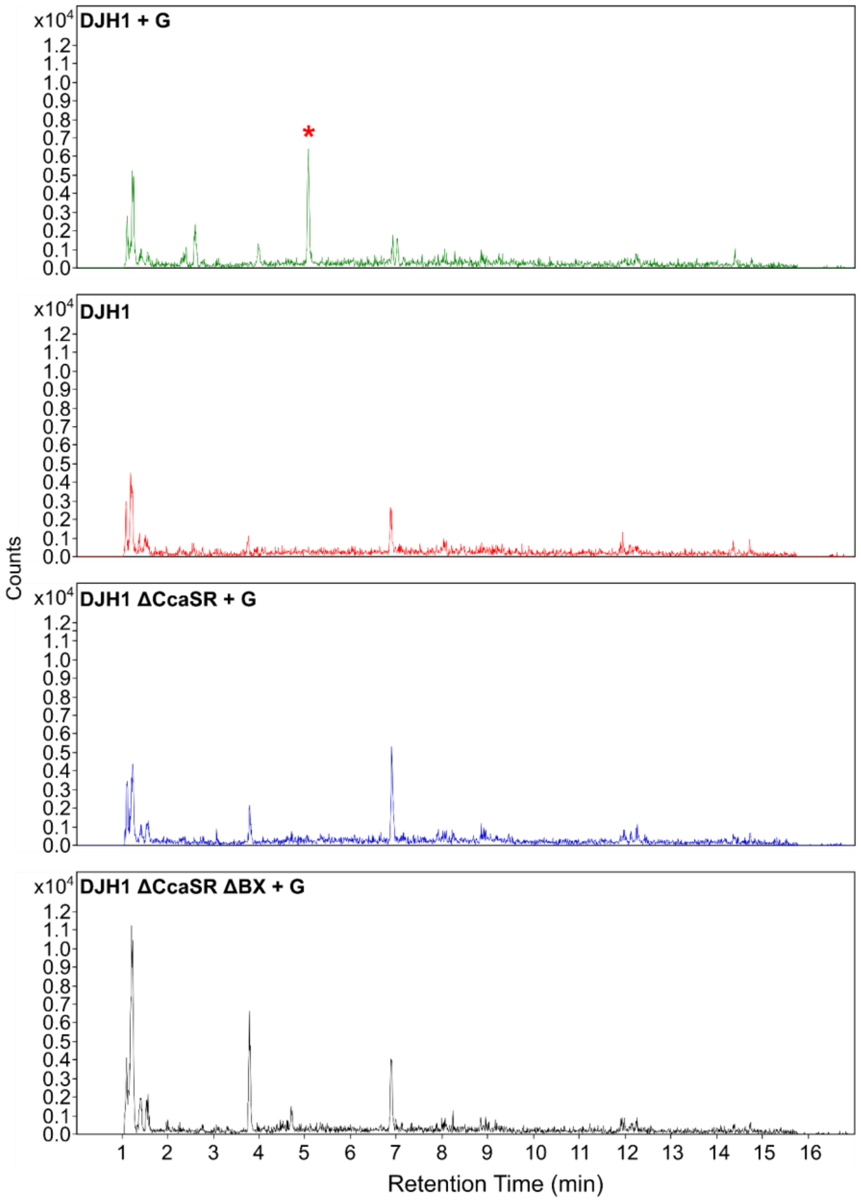

Supplementary Figure 11. LC Q-TOF MS traces for betalamic acid. Conditions and strains tested are the full betaxanthin production strain DJH1 under 100 µmol m^{-2} s^{-1} green light (DJH1 + G), DJH1 with no green light (DJH1), DJH1 with the pDJH019 plasmid bearing the betaxanthin pathway (pDJH019) but lacking CcaSR (pJTL257.2, pJTL269) under green light (DJH1 ΔCcaSR + G), and DJH1 lacking pDJH019, pJTL257.2, and pJTL269 under green light (DJH1 ΔCcaSR ΔBX + G). The peak marked with a red asterisk corresponds to betalamic acid.

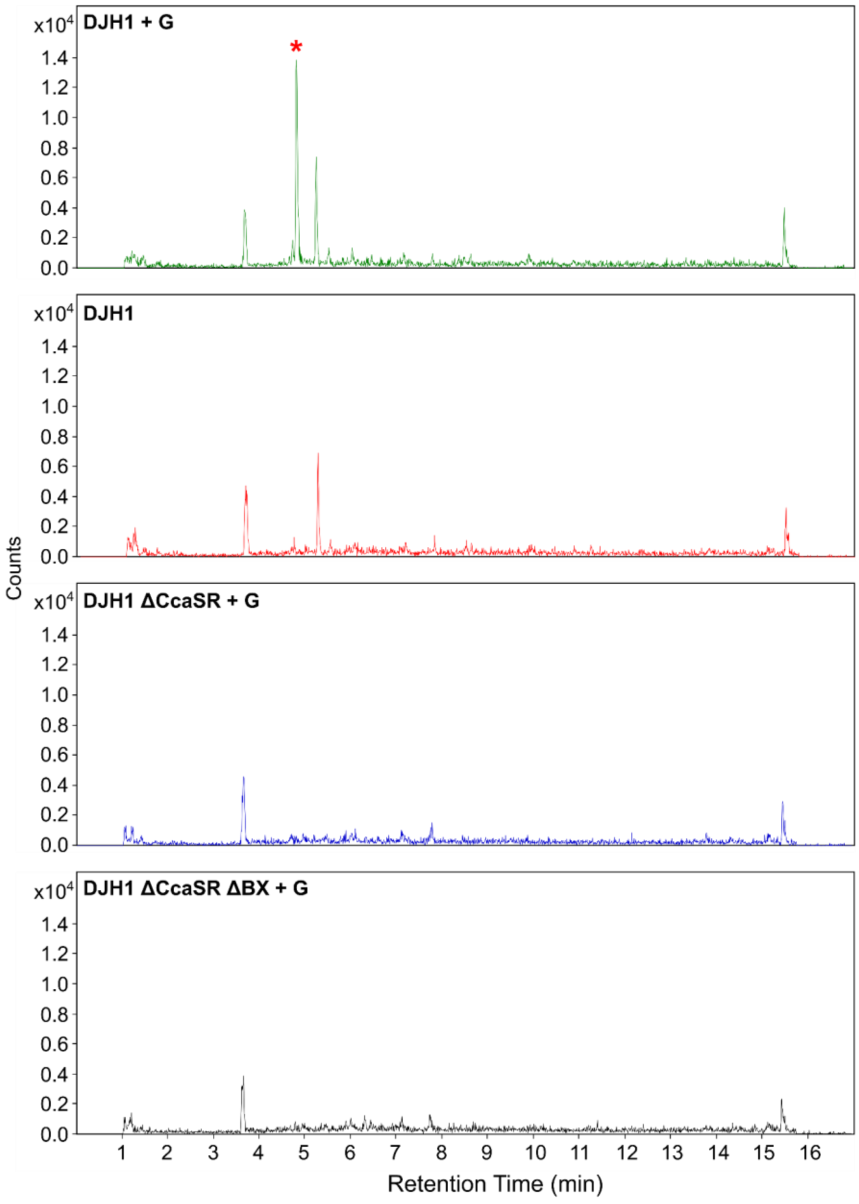

Supplementary Figure 12. LC Q-TOF MS traces for dopaxanthin. Conditions and strains tested are the full betaxanthin production strain DJH1 under 100 µmol m^{-2} s^{-1} green light (DJH1 + G), DJH1 with no green light (DJH1), DJH1 with the pDJH019 plasmid bearing the betaxanthin pathway (pDJH019) but lacking CcaSR (pJTL257.2, pJTL269) under green light (DJH1 ΔCcaSR + G), and DJH1 lacking pDJH019, pJTL257.2, and pJTL269 under green light (DJH1 ΔCcaSR ΔBX + G). The peak marked with a red asterisk corresponds to dopaxanthin.

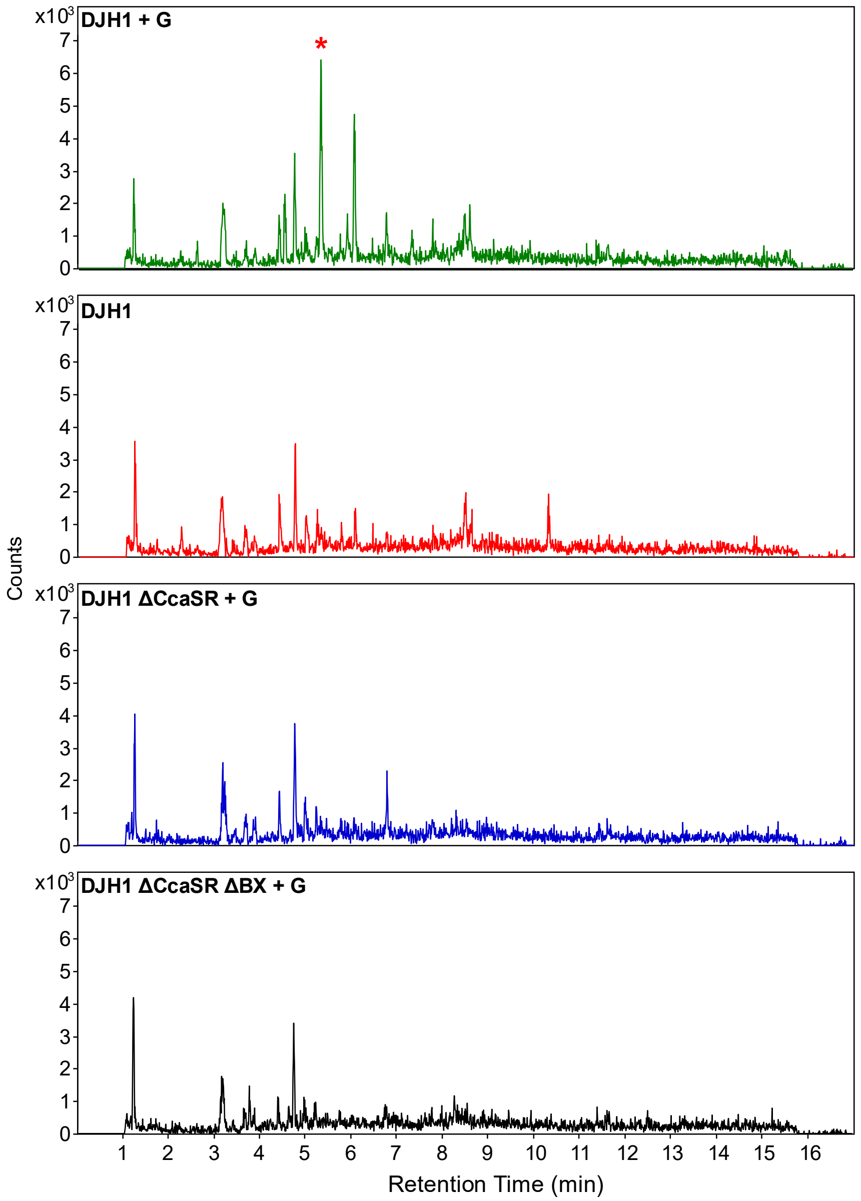

Supplementary Figure 13. LC Q-TOF MS traces for portulacaxanthin II. Conditions and strains tested are the full betaxanthin production strain DJH1 under 100 µmol m^{-2} s^{-1} green light (DJH1 + G), DJH1 with no green light (DJH1), DJH1 with the pDJH019 plasmid bearing the betaxanthin pathway (pDJH019) but lacking CcaSR (pJTL257.2, pJTL269) under green light (DJH1 ΔCcaSR + G), and DJH1 lacking pDJH019, pJTL257.2, and pJTL269 under green light

(DJH1 ΔCcaSR ΔBX + G). The peak marked with a red asterisk corresponds to portulacaxanthin II.

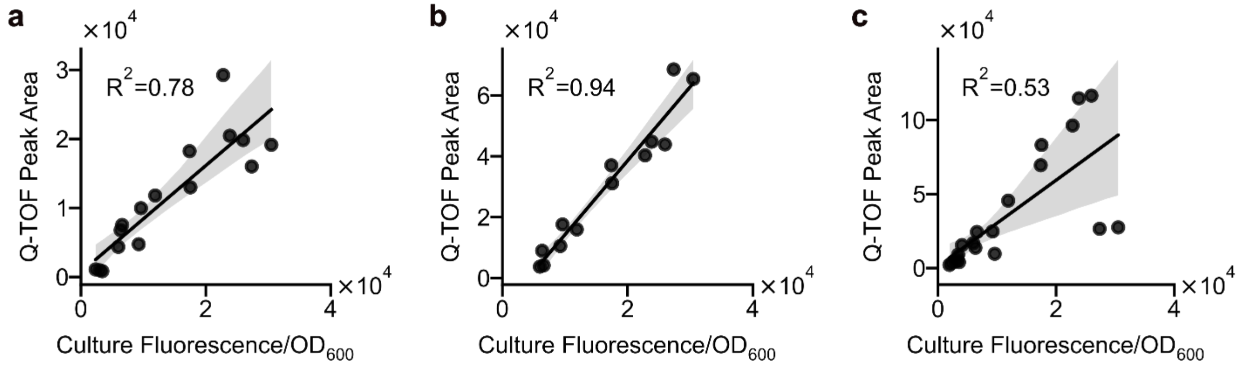

Supplementary Figure 14. Betaxanthin pathway products can be quantified by bacterial fluorescence. DJH1 cultures exposed to a variety of green light intensities were measured for bulk culture fluorescence (excitation 485 nm, emission 515 nm). Extracts of those cultures were analyzed for relative levels of (a) betalamic acid, (b) dopaxanthin, and (c) portulacaxanthin II, via LC Q-TOFMS as described in Methods.

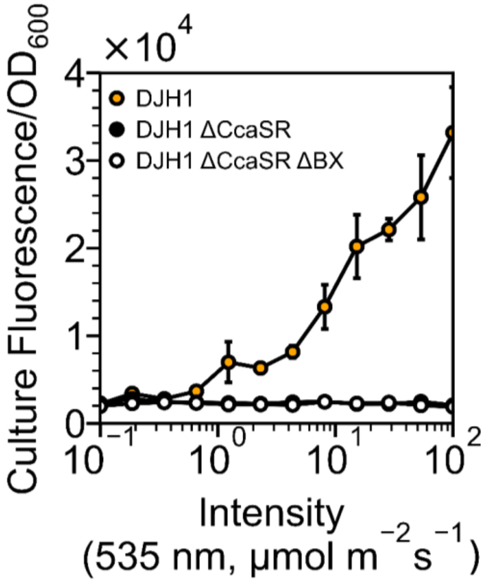

Supplementary Figure 15. Using $CcaSR_{stat}$ to optimize production of betaxanthins. DJH1 was grown in LPAs for 24 hours at 30°C and exposed to various intensities of green light. Cultures were measured for fluorescence at 485 nm excitation and 515 nm emission wavelengths. Error bars represent the mean of 3 replicates collected on the same day.

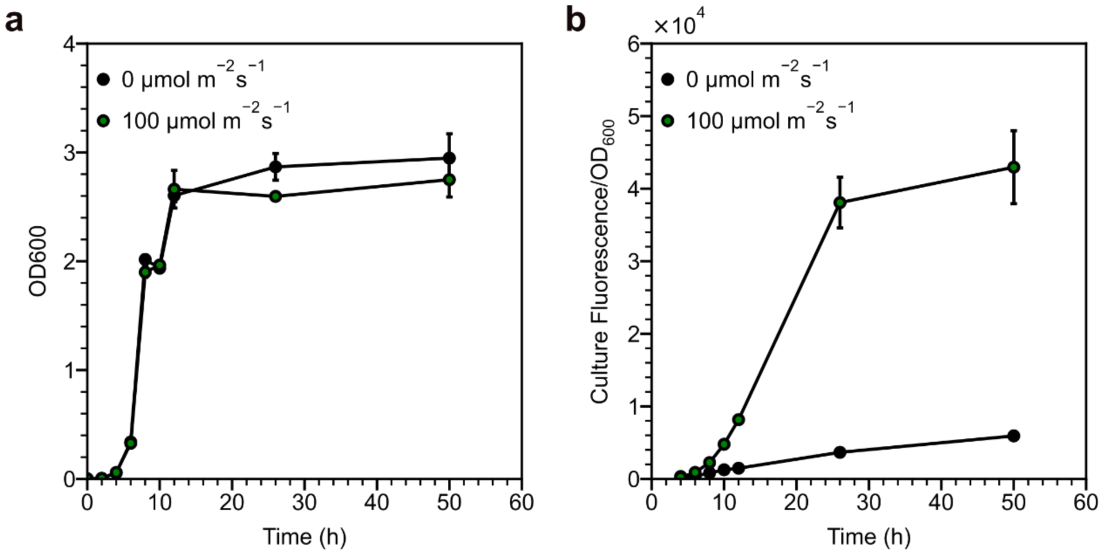

Supplementary Figure 16. Betaxanthins are primarily produced in stationary phase. DHJ1 culture (a) optical density and (b) betaxanthin product-dependent fluorescence (excitation 485 nm, emission 515 nm) over time. DJH1 cultures were started from exponential phase precultures diluted to an OD of 10^{-3}. Cultures were grown at 30°C in LPA devices. Error bars represent the means of 3 biological replicates collected on the same day.

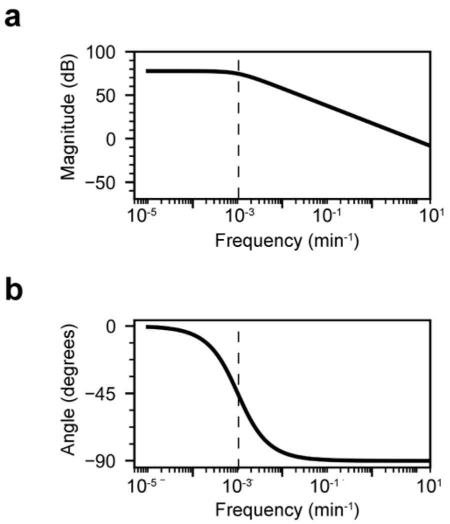

Supplementary Figure 17. Bode analysis of CcaSR$_{stat}$ response to periodic stationary phase activation. (a) Simulated magnitude and (b) phase shift of the CcaSR$_{stat}$ gene expression output in response to oscillatory green light inputs of different frequencies. To perform the simulations, the first-order CcaSR$_{stat}$ transfer function from Fig. 3F was used. Further information on the generation of the Bode plots is provided in the Methods. Below a period of T = 1,200 min (frequency of 8 x 10^{-4} min^{-1}), the magnitude of the Bode plot begins to decrease. Thus, periods less than 1,200 minutes (frequencies higher than 8 x 10^{-4} min^{-1}) are expected to result in attenuation of oscillatory CcaSR$_{stat}$ gene expression output signals. As a result of this analysis, we selected seven period lengths ranging from T = 0.08 minute, the shortest pulse achievable with our LPA device, to T = 1,000 minutes (dotted line on Bode plots) in Fig. 5B.

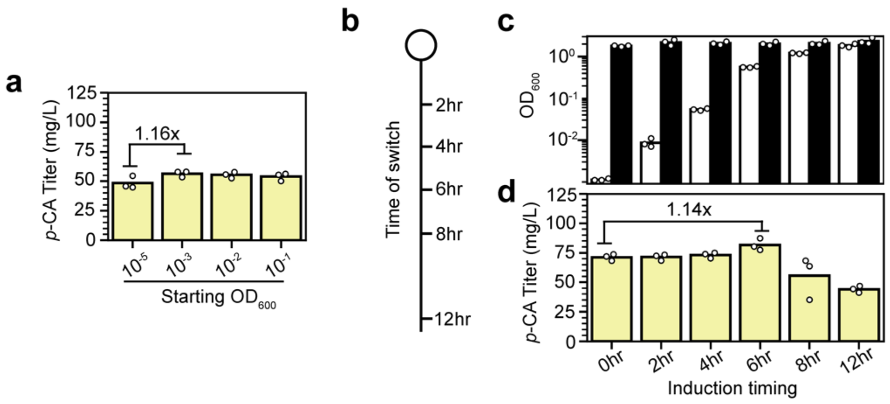

Supplementary Figure 18. Effect of initial culture density and *tal* induction timing on *p*-CA titer. (a) *p*-CA titer as a function of starting density of $CcaSR_{stat}$:*tal* cultures over the course of 72 h LPA fermentation. A static green light signal of 2 µmol m^{-2} s^{-1} was applied to all samples. Bars represent the arithmetic mean of *p*-CA titer of n = 3 independent biological replicates collected over 3 separate days. (b) Varying the induction timing of 72 h green light treatment in the *p*-CA fermentation. (c) OD_{600} values at the time of induction (white bar) and the final OD_{600} of cultures that were induced at different times. Bars represent the arithmetic mean of OD_{600} titer of n = 3 independent biological replicates collected over 3 separate days (d) Final *p*-CA titers for different induction times. Bars represent the arithmetic mean of *p*-CA titer of n = 3 independent biological replicates collected over 3 separate days. The optimal green light intensity of 7 µmol m^{-2} s^{-1} (Fig. 4C) was used. Although we find that induction at t = 6 h (OD_{600} = 0.26) results in a statistically significant increase in *p*-CA titer to 81.7 mg/L, the effect is small. This is likely due the fact that $CcaSR_{stat}$ has low expression in exponential phase regardless of light signal. The optimal induction timing of t = 6 h was used for subsequent experiments.

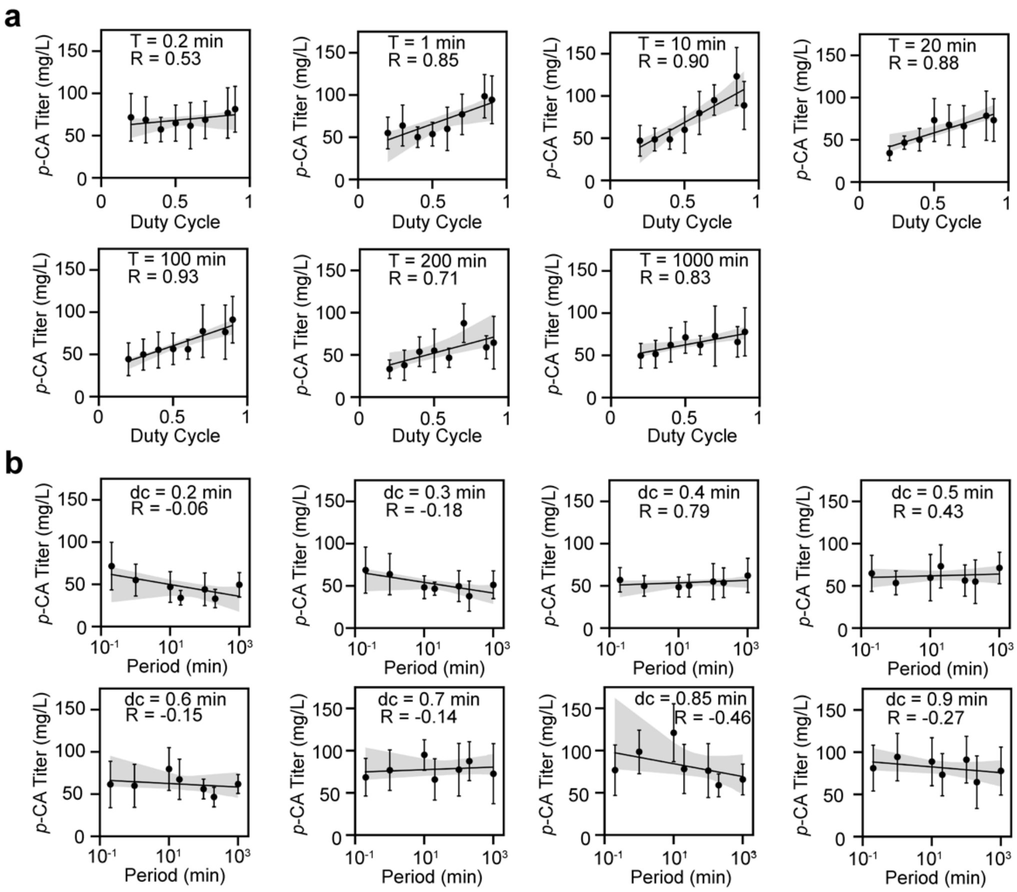

Supplementary Figure 19. Pairwise correlations between green light period, duty cycle, and *p*-CA titer. Linear regression between (a) duty cycle and *p*-CA titer at specified pulse period lengths along with (b) pulse period and *p*-CA titer at specified duty cycles. Pearson's correlation coefficient R is shown. Shaded region represents a 95% confidence interval of the linear regression. Data points and error bars represent the mean and standard deviation of *p*-CA titer across n = 5 independent biological replicates collected on 5 separate days.

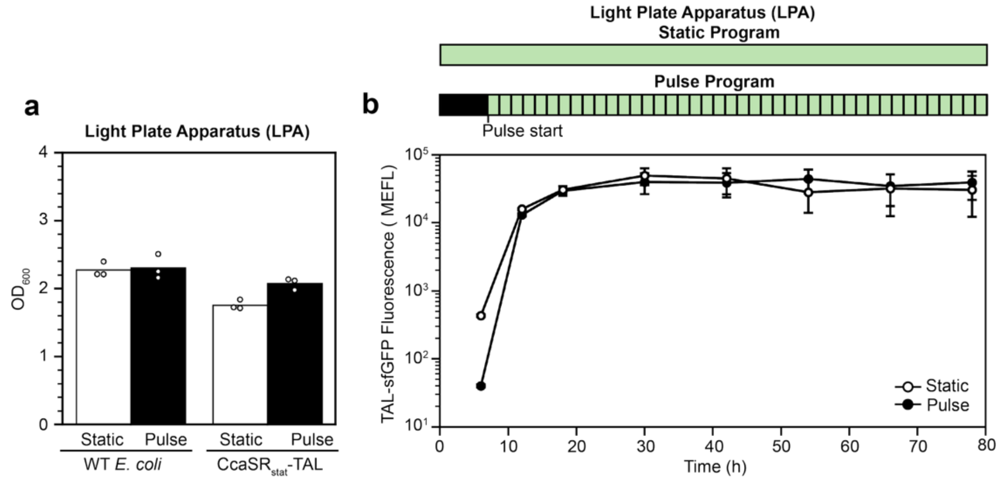

Supplementary Figure 20. TAL-mediated growth defects in static and pulsatile light conditions in Light Plate Apparatuses. (a) Growth defects of static (78 h of 7µmol m^{-2} s^{-1} green light) and pulse induction (6 h induction delay followed by 72 h of 7 µmol m^{-2} s^{-1} green light pulses with T = 10 min and dc = 85% patterns on background strain JTL1 and $CcaSR_{stat}$:*tal*. Bars represent the arithmetic mean of OD_{600} of *n* = 3 independent biological replicates collected on the same day and grown in the LPAs with the aforementioned light conditions at 30°C. (b) Measured TAL-sfGFP expression over the course of both the static and pulse induction fermentations in the LPA. Error bars represent the standard deviation of the mean of *n* = 6 independent biological replicates collected on the same day.

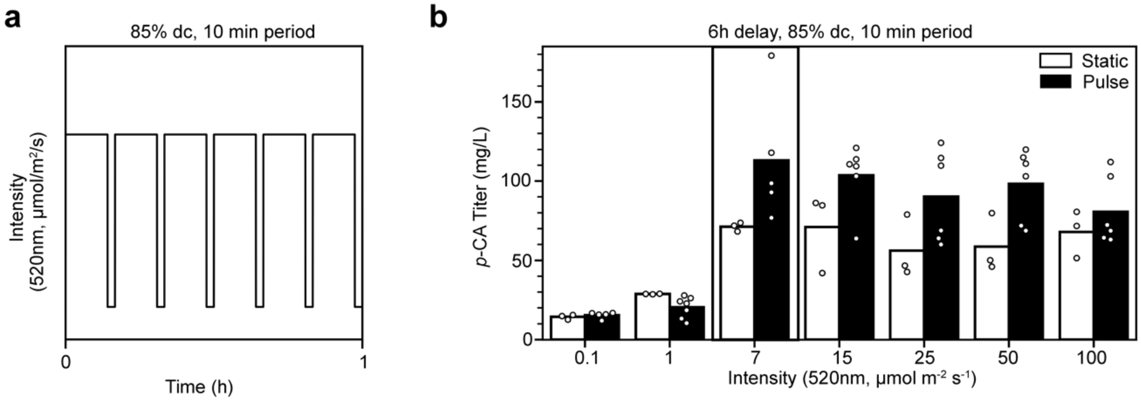

Supplementary Figure 21. Intensity optimization with optimal light pulsing conditions. (a) Light induction plot detailing the optimal pulse conditions (d.c. = 85% and T = 10 min.) over the course of 1 h. (b) Comparing results of static optimization (white bars) to optimization of green light intensity with optimal pulse conditions (black). Boxed data represents the optimal data from both the static and pulse optimizations in Fig. 4G and 5B where the bar of static data represents the mean of *n* =3 independent biological replicates collected on the same day and pulse data represents the mean of *n* = 5 independent biological replicates collected on 5 separate days. Non-boxed bars of static data represent the mean of *n* = 3 independent biological replicates of titers from Fig. 4G collected on the same day. Non-boxed bars of pulse data represent the mean of *n* = 6 independent biological replicates collected on the same day.

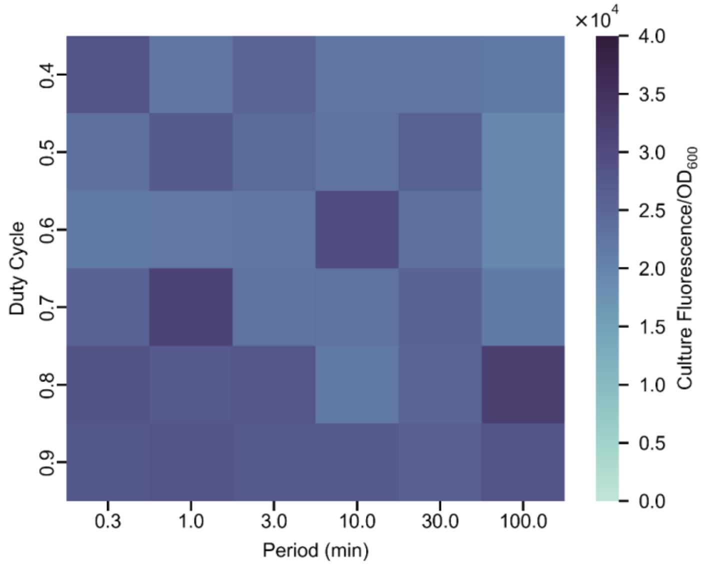

Supplementary Figure 22. Light pulsing does not improve betaxanthin pathway productivity. Relationship between T, d.c., and culture fluorescence divided by OD_{600}. Periodic green light treatment at 100 µmol m^{-2} s^{-1} took place over the course of the 24 h fermentation. Each colored square represents the mean of $n = 3$ replicates collected on the same day. There were no conditions for which measured culture fluorescence/OD_{600} had a statically significant increase compared to the value observed for static green light treatment at the same intensity with (one-tailed Welch's t-test, $p < 0.05$).

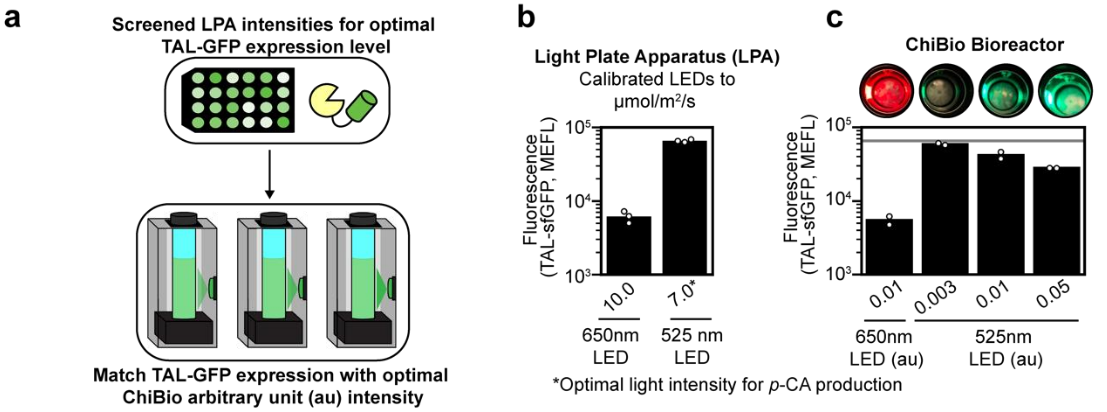

Supplementary Figure 23. Chi.Bio reactor calibrations. (a) Process of matching optimal TAL-sfGFP expression levels from the LPA device to the Chi.Bio reactors to find the optimal Chi.Bio green light intensity. (b) Red and green light responses of TAL-sfGFP at the end of a 72 h fermentation with static light signals. The green light response represents the TAL-sfGFP levels at the optimal LPA intensity (7 µmol m^{-2} s^{-1}). Bars represent the represent the arithmetic mean of TAL-sfGFP fluorescence across n = 3 independent biological data points collected on the same day. (c) Optimization of TAL-sfGFP expression at varying Chi.Bio green light intensities along with the red light response. Photographs of LEDs at each intensity are shown. Reported TAL-sfGFP levels are after t = 72 h with static light condition. The shaded region represents the standard deviation of the mean of optimal TAL-sfGFP LPA expression. Bars represent the arithmetic mean of TAL-sfGFP fluorescence across n = 2 independent biological replicate data points over two separate days.

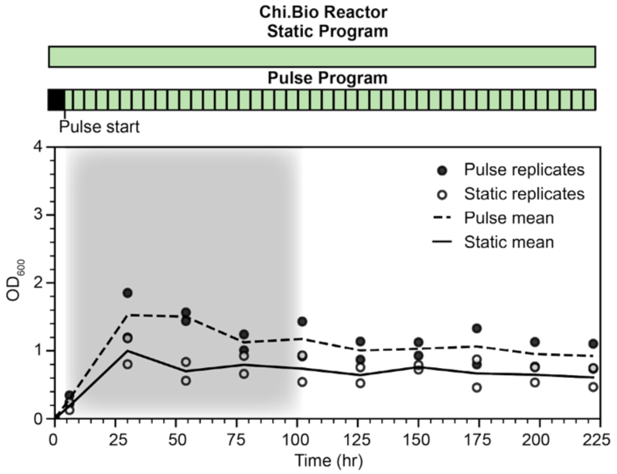

Supplementary Figure 24. Characterizing growth defects in Chi.Bio Reactors with static and dynamic light conditions. Growth in Chi.Bio reactors of the $CcaSR_{stat}$:*tal* strain with static (white) and optimal pulse (black) induction schemes. OD_{600} was measured from samples removed for *p*-CA quantification (Fig. 5D). Data points and mean lines for *n* = 2 independent biological reactor replicates collected over the same time course are shown as in Fig. 5d. The shaded gray region represents the time where a bolus of 5 mL M9 of 200 g L^{-1} glucose and 200 mg L^{-1} L-tyrosine was added to account for volume lost to sampling (Methods). The slight decrease in cell density is due to the slight dilution of reactor culture with the addition of the bolus while the cells are in stationary phase.

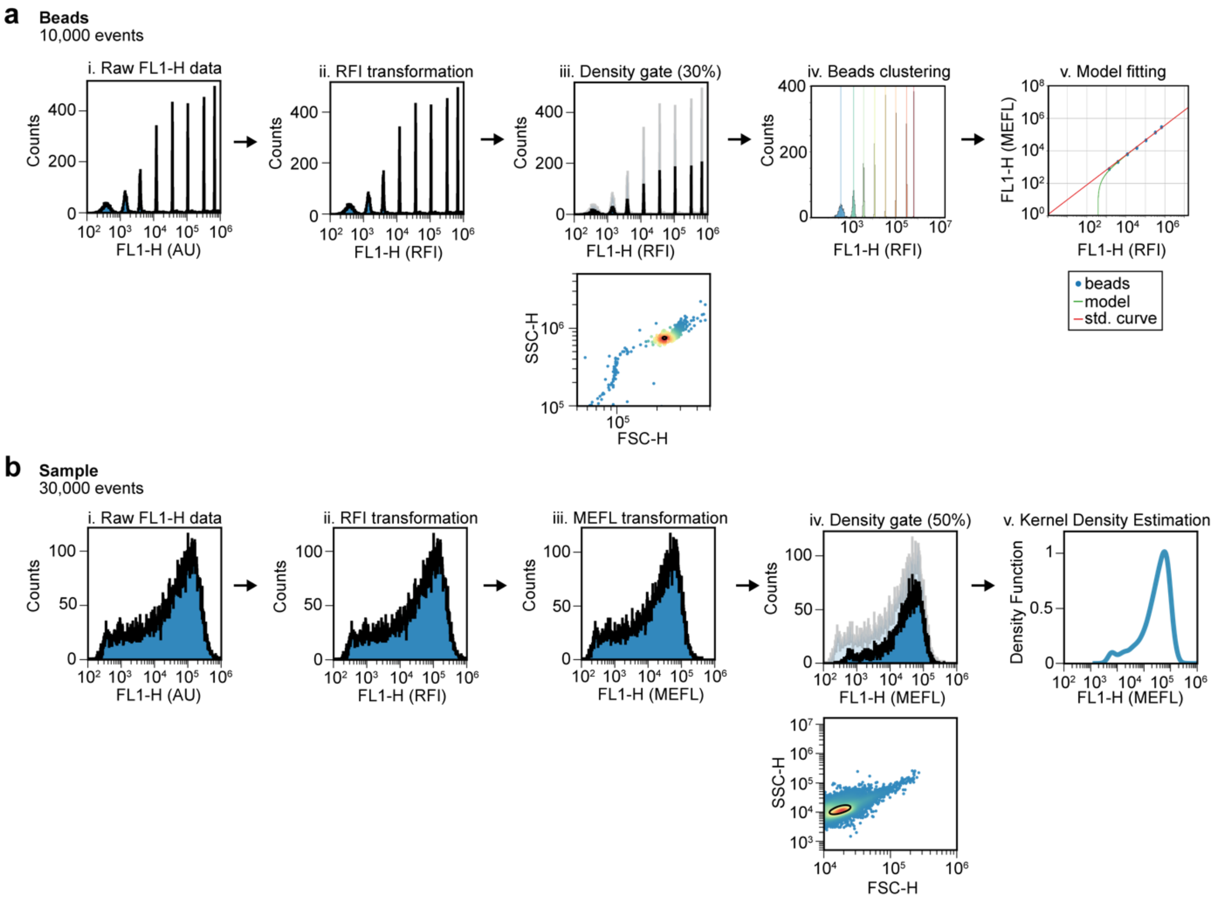

Supplementary Figure 25. Flow cytometry data calibration and analysis. (a) Measurement and processing of fluorescence calibration beads. Raw .fcs file FL1-H histograms (i) are transformed to relative fluorescence intensity (RFI) units (ii) and then density gated to 30% of total events (iii). Bead populations are then clustered to eight separate fluorescence intensity peaks. Different colors represent bead populations with distinct fluorescent values. (iv). The measured FL1-H RFI value of each cluster is then plotted against its standardized MEFL value to generate a calibration curve. This calibration curve is then utilized to generate a continuous model for fitting sample RFI data to MEFL[4]. (b) Sample processing. The raw sample .fcs file includes arbitrary units (AU) (i), which are initially transformed into RFI units (ii). The continuous model from the beads calibration in (a) is then used to transform the data into MEFL (iii). The sample histogram is then density gated to 50% off its total events. Redder colors

represent increasing event density (iv). We utilize the arithmetic mean of this population to calculate mean fluorescence of the sample. For histogram figures, we applied kernel density estimation (v) to generate smoothed outlines of the histograms, allowing one to view the distributions of several histograms on a single plot.

Supplementary References

1. Castillo-Hair, S. M., Baerman, E. A., Fujita, M., Igoshin, O. A. & Tabor, J. J. Optogenetic control of Bacillus subtilis gene expression. *Nat Commun* 10, 3099 (2019).
2. Schmidl, S. R., Sheth, R. U., Wu, A. & Tabor, J. J. Refactoring and Optimization of Light-Switchable *Escherichia coli* Two-Component Systems. *ACS Synth Biol* 3, 820–831 (2014).
3. Shao, B. et al. Single-cell measurement of plasmid copy number and promoter activity. *Nat Commun* 12, 1475 (2021).
4. Castillo-Hair, S. M. et al. FlowCal: A User-Friendly, Open Source Software Tool for Automatically Converting Flow Cytometry Data from Arbitrary to Calibrated Units. *ACS Synth Biol* 5, 774–80 (2016).
