# Supplementary material for: A stationary phase-specific bacterial green light sensor for enhancing metabolite production
Source: Nat Commun. 2025 Dec 24;17:1071. doi: 10.1038/s41467-025-67829-8 (PMC12852798; doi:10.1038/s41467-025-67829-8)
Supplement: Supplementary file 2 — Description of Additional Supplementary Information [file 41467_2025_67829_MOESM2_ESM.pdf]

Description of Additional Supplementary Files

File Name: Supplementary Data 1
Description: Primers and DNA sequences for analysis of ho1-pcyA operon stability.

File Name: Supplementary Data 2
Description: Strain details.

File Name: Supplementary Data 3
Description: Plasmid details.

File Name: Supplementary Data 4
Description: Genetic parts list.
